# *Haemoproteus tartakovskyi* and *Plasmodium relictum* (Haemosporida, Apicomplexa) differentially express distinct 18S rRNA gene variants in bird hosts and dipteran vectors

**DOI:** 10.1186/s13071-025-06696-0

**Published:** 2025-02-20

**Authors:** Josef Harl, Tanja Himmel, Mikas Ilgūnas, Carolina Romeiro Fernandes Chagas, Julia Matt, Nora Nedorost, Tatjana A. Iezhova, Gediminas Valkiūnas, Herbert Weissenböck

**Affiliations:** 1 Department of Pathology, Medical University Vienna, Währinger Guertel 18–20, 1090 Vienna, Austria; 2 Department for Biological Sciences and Pathobiology, University of Veterinary Medicine Vienna, Veterinaerplatz 1, 1210 Vienna, Austria; 3 Nature Research Centre, Akademijos 2, 08412 Vilnius, Lithuania

**Keywords:** Chromogenic in situ hybridization, Histology, *Culex quinquefasciatus*, *Culicoides nubeculosus*, Sporogony

## Abstract

**Background:**

Most mammalian *Plasmodium* species possess distinct 18S ribosomal RNA (rRNA) gene copies, which are differentially expressed in vertebrate hosts and mosquito vectors. Although similar sequence patterns were found in avian haemosporidian parasites, expression patterns have not been studied yet. This study aimed to test whether 18S variants of *Plasmodium relictum* SGS1 and *Haemoproteus tartakovskyi* SISKIN1 are expressed differentially in bird hosts and dipteran vectors using real-time quantitative polymerase chain reaction (qPCR) and chromogenic in situ hybridization (CISH).

**Methods:**

Eurasian siskins (*Spinus spinus*) experimentally infected with *P. relictum* SGS1 and naturally infected with *H. tartakovskyi* SISKIN1 were used. *Culex quinquefasciatus* mosquitoes (SGS1) and *Culicoides nubeculosus* biting midges (SISKIN1) were fed on the blood of infected birds and maintained for several days to allow for the development of oocysts and sporozoites. Total RNA was extracted from bird blood and a subset of the dipteran vectors during each stage of parasite development, followed by qPCRs specifically targeting distinct 18S variants of the two parasites. Organs of the donor birds and whole bodies of the vectors were examined histologically using CISH by targeting different 18S variants of the parasites.

**Results:**

*Plasmodium relictum* SGS1 expressed two 18S variants in bird blood and mosquitoes, but their expression levels were reversed in birds and vectors, with one variant being preferentially expressed over the other. Using CISH, oocysts were stained with probes targeting both 18S variants, but sporozoites could not be detected, suggesting a suboptimal development of the parasites. *Haemoproteus tartakovskyi* SISKIN1, which features three distinct *18S* variants, expressed one 18S variant in bird blood and two variants in the biting midges, while no signals were detected for the third variant. The results were corroborated by CISH, but surprisingly, some oocysts were also stained by the probe targeting the third variant.

**Conclusions:**

The results indicate that distinct 18S variants of the two parasite species are differentially expressed in bird hosts and vectors. Moreover, for the first time, we provide visualizations of avian haemosporidian oocysts in tissue sections of the vectors, with the discovery of extraintestinal development of oocysts in SISKIN1-infected biting midges.

**Graphical Abstract:**

**Figure:**
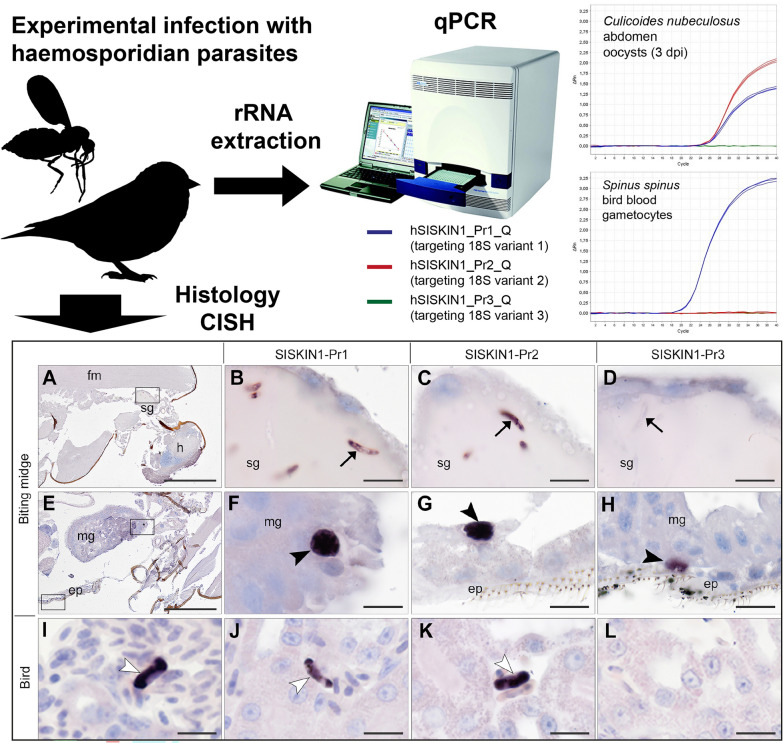

**Supplementary Information:**

The online version contains supplementary material available at 10.1186/s13071-025-06696-0.

## Background

Human, simian, and rodent *Plasmodium* species are considered exceptional within the domain of Eukaryota because their ribosomal units evolve according to a birth-and-death model under strong purifying selection. *Plasmodium* species feature only four to eight nuclear ribosomal units per haploid genome, which are located on different chromosomes and assumed to accumulate mutations independently [1]. Moreover, experimental studies on the 18S ribosomal RNA (rRNA) genes of *Plasmodium berghei*, *Plasmodium yoelii*, *Plasmodium falciparum*, *Plasmodium cynomolgi*, and *Plasmodium vivax* have proven differential expression of distinct ribosomal units in vertebrate hosts and dipteran vectors [2–6]. So-called A-type ribosomal DNA (rDNA) units are expressed by “asexual stages” (tissue and blood stages, including micro- and macrogametocytes) in the vertebrate host, whereas the S-type units are expressed by sporozoites in the mosquito vectors [4, 7]. A third class of 18S rRNA types, the so-called O-type variant, was found to be expressed by oocysts of *P. vivax* in the mosquito midgut shortly after fertilization [5]. Qi et al. [6] characterized another 18S rRNA variant in *P. yoelii*, which is mainly expressed by ookinetes and young oocysts, and termed it D-type as a subtype of the S-type genes.

Fang and McCutchan [3] showed that temperature is a crucial factor driving the transcription of distinct 18S variants in laboratory cultures of *P. falciparum*, with A-type variants preferentially being expressed at higher temperatures (vertebrate host) and S-type variants at lower temperatures (mosquito vector). Since parasites entering the mosquito host face a severe drop in glucose concentration compared to the vertebrate host, glucose is another potential regulator of transcription; asexual blood stages of *P. falciparum* subjected to glucose starvation decreased the expression of A-type variants and increased the expression of S-type variants [3]. Strongly diverged 18S variants were found in most human, rodent, and simian *Plasmodium* species except for *Plasmodium malariae*. The genetic distances between A-type and S-type 18S rRNA genes are up to 10.9% in *P. vivax* and related simian *Plasmodium* species, 11.3% in *P. berghei* and other members of the subgenus *Vinckeia*, 12.9% in *P. falciparum* and other members of the subgenus *Laverania*, and 17.0% and 17.6% in *Plasmodium ovale curtisi* and *Plasmodium ovale wallikeri*, respectively [8]. Despite the exceptional features and expression patterns of 18S rRNA genes found in mammalian *Plasmodium* species, ribosomal genes of avian haemosporidian parasites have been widely neglected*.* Only recently, Harl et al. [8, 9] published 18S rRNA gene sequences of almost 50 avian *Plasmodium*, *Haemoproteus*, and *Leucocytozoon* lineages and found that avian haemosporidian parasites also feature distinct copies of the 18S rRNA genes to varying degrees. The genetic distances between 18S variants were low in *Plasmodium relictum* SGS1, at 2.3%, but high in *Plasmodium matutinum* LINN1 and *Plasmodium elongatum* GRW06, at 10.8% and 14.9%, respectively, which is comparable to the distance found in mammalian *Plasmodium* species. More strongly diverged 18S rRNA variants, with distances ranging from 16.9% to 18.5%, were only found in parasites of the *Leucocytozoon toddi* group, whose lineages are exclusively found in accipitriform raptors [8]. By contrast, most *Leucocytozoon* and *Haemoproteus* lineages have only slightly diverged 18S rRNA variants clustering in reciprocally monophyletic subclades. The only exception so far is *Haemoproteus tartakovskyi* SISKIN1, which features two similar 18S variants diverged by 6.3%, and a third one diverged from the latter by 18.7% to 21.5% [8]. *Haemoproteus tartakovskyi* SISKIN1 is the only *Haemoproteus* species whose genome was sequenced [10], although only sequence contigs were published and no assembled chromosomes.

The presence of distinct 18S variants in *P. relictum* SGS1, *H. tartakovskyi* SISKIN1, and other avian haemosporidian parasites was confirmed previously [8, 9], but their expression patterns have not yet been studied. This study aimed to experimentally test whether distinct 18S rRNA variants of *P. relictum* SGS1 and *H. tartakovskyi* SISKIN1 are differentially expressed in bird hosts and dipteran vectors as was shown previously for several mammalian *Plasmodium* species [2–6]. We performed experimental infections using the latter two parasite lineages and compared the expression of their 18S rRNA variants in bird hosts and dipteran vectors using real-time quantitative polymerase chain reaction (qPCR) and chromogenic in situ hybridization (CISH). *Haemoproteus tartakovskyi* SISKIN1 was chosen because it is the only *Haemoproteus* species known to feature very distinct 18S rRNA gene variants and it completes sporogony in the biting midge *Culicoides nubeculosus*, of which lab colonies were established at the Nature Research Centre (NRC) in Vilnius. *Plasmodium relictum* SGS1 was selected because it is one of the most widely distributed avian *Plasmodium* species, features distinct 18S rRNA variants, and completes sporogony in *Culex pipiens* and *Culex quinquefasciatus* mosquitoes, of which lab colonies were available at the NRC in Vilnius.

## Methods

### Experimental infection of birds and vectors

Eurasian siskins (*Spinus spinus*) and domestic canaries (*Serinus canaria*) were purchased commercially from licensed breeders at Vogelhandel Van der Wegen (Steenbergen, Netherlands). Experimental infections of birds were carried out at the NRC in Vilnius, Lithuania, where the birds were kept in a licensed vivarium in a vector-free room under controlled conditions (20 ± 1 °C; 50–60% relative humidity; natural photoperiod) for the duration of the experiment until they were euthanized. The permits were obtained from the Ethical Commission of the Baltic Laboratory Animal Science Association and the Lithuanian State Food and Veterinary Office.

#### *Plasmodium relictum* SGS1

A commercially purchased domestic canary and two Eurasian siskins were used for the experimental infections. To confirm that the birds were free of haemosporidian parasites, blood was taken from the brachial vein and subjected to microscopy and PCR-based molecular analysis targeting the cytochrome b (*CytB*) barcode region as described previously [11]. Genetically characterized frozen blood samples of *P. relictum* SGS1 from the cryo-bank of the NRC Vilnius were used to infect the canary by blood inoculation as described previously [12, 13]. After parasitemia developed, the canary’s blood was used to infect the two Eurasian siskins as described previously [14]. Drops of blood from the siskins were stored in 70% ethanol (EtOH) at 4 °C for RNA extraction. After parasitemia developed, *Cx. quinquefasciatus* mosquitoes were allowed to feed on the siskins in an experimental setup as described previously [15]. Blood-fed female mosquitoes were taken from the experimental cages using an aspirator, placed in separate small insect cages, and maintained at a mean laboratory temperature of 27 ± 1.3 °C, relative humidity of 55 ± 5%, and controlled light–dark photoperiod of 17:7 h for up to 17 days post-infection (dpi). To confirm that the infection was successful, some mosquitoes were dissected in a drop of 0.9% normal saline at 1 dpi (9 individuals), 10 dpi (7 ind.), and 17 dpi (8 ind.) to check for ookinetes, oocysts, and sporozoites, respectively. For RNA extractions, mosquitoes were sampled at 9–10 dpi (12 ind.; oocyst stage) and 16–17 dpi (14 ind.; sporozoite stage). The mosquitoes were anesthetized by putting them into tubes with cotton pads soaked in 96% EtOH for several minutes. The wings and legs of the mosquitoes were removed under a binocular stereoscopic microscope and the bodies were divided into two parts (thorax/head and abdomen) before they were stored separately in 1.5 ml tubes with 70% ethanol. For histology and CISH, mosquitoes were sampled at 9–10 dpi (13 ind.) and at 16 dpi (14 ind.), fixed individually in 10% neutral buffered formalin for 24 h, and then stored in 70% EtOH. In addition, three uninfected mosquitoes were collected as negative controls, one of which was used for RNA extraction and two for histological analyses. Blood samples of the two infected donor siskins were stored in 1.5 ml tubes with 70% EtOH for RNA extraction. All samples were stored at 4 °C until shipment to Vetmeduni.

#### *Haemoproteus tartakovskyi* SISKIN1

Naturally infected wild Eurasian siskins were caught with mist nets in Ventės Ragas. Blood was collected with heparinized microcapillary tubes by puncturing the brachial vein and stored in SET buffer (0.15 M NaCl, 0.05 M Tris, 0.5 M EDTA, pH 8.0) for molecular analysis. Blood drops were used to prepare blood films, which were fixed in methanol and stained with Giemsa. The blood films were inspected microscopically to confirm the presence of *H. tartakovskyi* gametocytes. Two Eurasian siskins, which were naturally infected with *H. tartakovskyi*, were used for experimental infections of *Cu. nubeculosus* biting midges. Drops of blood from the infected birds were stored in 70% EtOH for RNA extraction. Colony-reared *Cu. nubeculosus* were allowed to take blood meals on the two Eurasian siskins as described previously [16]. The exposed biting midges were maintained at 27 ± 1.3 °C, relative humidity of 55 ± 5%, and a controlled light–dark photoperiod of 17:7 h. To confirm that the infection was successful, individual biting midges were dissected in a drop of 0.9% normal saline to examine midgut contents for ookinetes (9 h post-infection; 2 ind.) and oocysts (4 dpi, 1 ind.), and salivary glands for sporozoites (7 dpi, 1 ind.). For the RNA extractions, biting midges were sampled at 3–5 dpi (7 ind.) and at 7 dpi (6 ind.). Their bodies were divided into head/thorax and abdomen, which were then stored separately in 1.5 ml tubes with 70% EtOH. For histological analyses and CISH, biting midges were also sampled at 3–5 dpi (5 ind.) and at 7 dpi (6 ind.), fixed individually in 10% neutral buffered formalin for 24 h, and then stored in 70% EtOH. As negative controls, five uninfected biting midges were collected, two of which were used for RNA extractions, and three for histological analyses. All samples were stored at 4 °C until shipment to Vetmeduni.

### RNA extractions

RNA extractions were performed on body parts of experimentally infected mosquitoes and biting midges, and blood of the donor birds. The RNA extraction procedure for the dipteran vectors and blood samples was identical. Before the RNA extraction, mosquito parts and pellets of coagulated blood from the donor birds (c. 2 mm in diameter) were dried by exposure to air for 5 min. Insect body parts and blood pellets were then transferred into RNase-free 2 ml tubes with steel forceps (sterilized by flaming) and homogenized in 1 ml innuSOLV reagent (Analytik Jena, Jena, Germany) for 5 min. After adding 0.2 µl 1-bromo-3-chloropropane (BCP; Sigma-Aldrich, St. Louis, MO, USA) as phase separation agent, the tubes were vortexed for 10 s and incubated on ice for 10 min. Then the tubes were centrifuged at 10,000 rpm and 4 °C for 5 min to separate the phases. The upper colorless phase containing the RNA (c. 50% of the volume) was transferred into new centrifuge tubes, equal volumes of isopropanol were added, and the samples were incubated for 15 min at 4 °C. The supernatant was removed, and the pellet was washed twice using 1 ml 70% EtOH by centrifuging at 13,000 rpm for 10 min and discarding the EtOH by pipetting. The RNA pellet was dried for 5 min by exposure to air, and the RNA was dissolved in 50 µl RNase-free water. The RNA concentration and 260/230 and 260/280 ratios of each sample were measured on a DS-11 spectrophotometer (DeNovix, Wilmington, DE, USA) calibrated with RNase-free water. The RNA quality was checked by loading 2 µl RNA extract mixed with 2 µl Gel-Loading Buffer II (Thermo Fisher, Waltham, MA, USA) on 1% agarose gels stained with ROTI^®^GelStain (Carl Roth GmbH & Co. KG, Karlsruhe, Germany). DNase digestion and clean-up were performed with the Zymo RNA Clean & Concentrator Kit (Zymo Research, Orange, CA, USA). Each 40 µl of the RNA extracts were mixed with 5 µl DNase I (1u/µl) and 5 µl DNA digestion buffer and incubated at room temperature for 15 min. A total of 100 µl RNA binding buffer and 150 µl 100% EtOH were added to each sample, mixed, and transferred to Zymo-Spin™ IC Columns in collection tubes. A total of 400 µl RNA Prep Buffer, 700 µl RNA Wash Buffer, and 400 µl RNA Wash Buffer were added to the columns and separated by centrifugation steps at 13,000 rpm for 30 s, and the flow-through discarded. In the last centrifugation step, RNA was eluted with 20 µl RNase-free water, and the RNA concentration and 260/230 and 260/280 ratios were measured again. Reverse transcription was performed with the iScript cDNA [complementary DNA] Synthesis kit (Bio-Rad Laboratories, Hercules, CA, USA) in 0.2 ml tubes using 15 µl RNA extract, 4 µl iScript reaction mix, and 1 µl iScript reverse transcriptase. The samples were treated at 25 °C for 5 min (priming), 46 °C at 20 min (reverse transcription), and 96 °C for 1 min (RT inactivation). Finally, 80 µl RNase/DNase-free water was added to bring to volumes of 100 µl, and the DNA content was measured with the DS-11 spectrophotometer. All samples were stored at −20 °C.

### Primers and probes

#### *Plasmodium relictum* SGS1

**Fig. 1:**
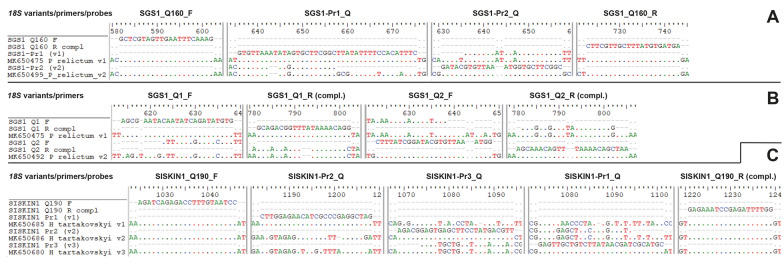
Alignment schemes showing primer and probe binding sites in the *Plasmodium relictum* SGS1 (**A**, **B**) and *Haemoproteus tartakovskyi* SISKIN1 (**C**) 18S rRNA gene sequences. **A** Primers and probes used in the *P. relictum* SGS1 TaqMan qPCR assay. **B** Variant-specific primers used in the dye-based *P. relictum* SGS1 qPCR assay. **C** Primers and probes used in the *H. tartakovskyi* SISKIN1 TaqMan qPCR assay

Based on previously published, almost complete 18S rRNA gene sequences from 32 clones of *P. relictum* SGS1 (MK650473-83, MK650484-94, and MK650495-50) [8], primers and probes targeting the two distinct 18S rRNA variants were designed for the PCR assays (standard PCR and qPCR) and CISH. The primers flank the sequence region most strongly diverged between the two 18S variants, which was also selected as the target region for the TaqMan probes for qPCR and the probes for CISH. The primers SGS1_Q160_F and SGS1_Q160_R, starting approximately 635 base pairs (bp) from the 5′-end of the *18S*, amplify fragments of 159–161 bp (depending on the 18S variant). The qPCR probes were placed between the two primers and targeted each one of the two 18S variants in the 5′–3′ direction. The probe SGS1-Pr1_Q matches 28 of 32 clones and SGS1-Pr2_Q matches four of 32 clones published by Harl et al. [8]. Both TaqMan probes were modified with FAM at 5′ and TAMRA at 3′. The probes for CISH, SGS1-Pr1_ISH and SGS1-Pr2_ISH, were identical to the TaqMan probes but reverted since the aim was to directly target the rRNA of the 18S rRNA gene. The CISH probes were labeled with digoxigenin at 3′. Since the qPCRs with the TaqMan probe SGS1-Pr1_Q were not successful, probably due to the length and low GC content of the probe, we additionally designed variant-specific primers for a dye-based qPCR assay. The primers SGS1_Q1_F and SGS1_Q1_R target the same 18S variant as SGS1-Pr1_Q/SGS1-Pr1_ISH, and SGS1_Q2_F and SGS1_Q2_R target the same 18S variant as SGS1-Pr1_Q/SGS1-Pr1_ISH. Primers and probes for the *P. relictum* SGS1 experiments are listed in Table 1. Schemes showing the primer and probe binding sites are provided in Fig. 1.

**Table 1:** Primers and probes used for analysis of *P. relictum* SGS1 differential 18S rRNA gene expression

Primer/probe	Description	Targeted *18S* variant^a^	Primer/probe sequences (5′–3′)	Annealing temp (°C)
SGS1_Q160_F	Forward primer	v1, v2	GCT CGT AGT TGA ATT TCA AAG	53.6
SGS1_Q160_R	Reverse primer	v1, v2	TCA TCA CAT AAA GCA ACG AAG	54.6
SGS1-Pr1_Q	TaqMan qPCR probe	v1	FAM-GTG TTA AAT ATA GTG CTT CGG CTT ATA TTT TCC ACA TTT C-TAMRA	63.0
SGS1-Pr2_Q	TaqMan qPCR probe	v2	FAM-GAT ACG TGT TAA ATG GTG CTT CGG C-TAMRA	62.9
SGS1-Pr1_ISH	CISH probe	v1	GAA ATG TGG AAA ATA TAA GCC GAA GCA CTA TAT TTA ACA C-digoxigenin	63.0
SGS1-Pr2_ISH	CISH probe	v2	GCC GAA GCA CCA TTT AAC ACG TAT C-digoxigenin	62.9
SGS1_Q1_F	Forward dye-based qPCR primer	v1	AGC GAA TAC AAT ATC AGA TAT GTG	54.8
SGS1_Q1_R	Reverse dye-based qPCR primer	v1	CCT GTT TTA TAA ACC GTC TGC	55.2
SGS1_Q2_F	Forward dye-based qPCR primer	v2	CTT TAT CGG ATA CGT GTT AAA TGG	56.0
SGS1_Q2_R	Reverse dye-based qPCR primer	v2	TTA GCT GTT TTA AAC TGT TTG CT	55.5

^a^v1: variant 1; v2: variant 2

#### *Haemoproteus tartakovskyi* SISKIN1

Based on previously published *H. tartakovskyi* SIKSIN1 18S rRNA gene sequences obtained from 10 18S clones (MK650680-89) [8] and genomic contigs 65 and 602 of BioProject PRJNA309868 [10], primers and probes targeting three distinct 18S variants were designed for the PCR assays and CISH. The primers SISKIN1_Q190_F and SISKIN1_Q190_R, starting approximately 1000 bp from the 5′-end of the 18S, amplify fragments of 186 bp, 189 bp, and 215 bp, respectively, and bind all three 18S variants without mismatches. The probes for the qPCRs were placed between the two primers and targeted each one of the three 18S variants of *H. tartakovskyi* SISKIN1 in the 5′-3′ direction. The probe SISKIN1-Pr1_Q matches one clone (MK650685), SISKIN1-Pr2_Q matches two clones (MK650686, MK650689) and contig 602 (BioProject PRJNA309868), and SISKIN1-Pr3_Q matches seven clones (MK650680–84, MK650687–88) and contig 65 (BioProject PRJNA309868). The CISH probe sequences of SISKIN1-Pr1_ISH, SISKIN1-Pr2_ISH, and SISKIN1-Pr3_ISH were identical to the TaqMan probes but reverted to primarily bind the rRNAs of the ribosomes. The CISH probes were labeled with Digoxigenin at 3′. Primers and probes for the *H. tartakovskyi* SISKIN1 experiments are listed in Table 2. Schemes showing the primer and probe binding sites are provided in Fig. 1.

**Table 2:** Primers and probes used for analysis of *H. tartakovskyi* SISKIN1 differential 18S rRNA expression

Primer/probe	Description	Targeted 18S variant^a^	Primer/probe sequences (5′–3′)	Annealing temp (°C)
SISKIN1_Q190_F	Primer	v1, v2, v3	AGA TCA GAG ACC TTT GTA ATC C	55.0
SISKIN1_Q190_R	Primer	v1, v2, v3	CCC AAA ATC TCG GAT TTC TC	54.1
SISKIN1-Pr1_Q	TaqMan qPCR probe	v1	FAM-CTT GGA GAA CAT CGC CCG AGG CTA G-TAMRA	64.9
SISKIN1-Pr2_Q	TaqMan qPCR probe	v2	FAM-AGA CGG AGT GAG CTT CCT ATG ACG TT-TAMRA	65.4
SISKIN1-Pr3_Q	TaqMan qPCR probe	v3	FAM-GAG TTG CTG TCT TAT AAC GAT CGC ATG C-TAMRA	64.7
SISKIN1-Pr1_ISH	CISH probe	v1	CTA GCC TCG GGC GAT GTT CTC CAA G-Digoxigenin	64.9
SISKIN1-Pr2_ISH	CISH probe	v2	AAC GTC ATA GGA AGC TCA CTC CGT CT-Digoxigenin	65.4
SISKIN1-Pr3_ISH	CISH probe	v3	GCA TGC GAT CGT TAT AAG ACA GCA ACT C-Digoxigenin	64.7

^a^v1: variant 1, v2: variant 2, v3: variant

### PCRs for parasite detection in cDNA samples

To confirm the presence of parasite rRNA in donor birds and vectors, all cDNA samples obtained from RNA extracts of insect body parts and bird blood were subjected to standard PCR with the primers SGS1_Q160_F/SGS1_Q160_R and SISKIN1_Q190_F/SISKIN1_Q190_R. The PCRs were conducted with the KAPA2G Fast HotStart ReadyMix polymerase kit (Sigma-Aldrich, St. Louis, MO, USA) in 25 µl volumes containing 12.5 µl KAPA2G Fast HotStart ReadyMix, 7.5 µl nuclease-free water, 1 µl 1.5 mM MgCl_2_, each 1 µl forward and reverse primer (10 mM), and 2 µl template. The PCRs started with an initial denaturation for 2 min at 94 °C, followed by 35 cycles with 15 s at 94 °C, 15 s at 50 °C, 15 s at 72 °C, and a final extension for 10 min at 72 °C. The PCR products were visualized on 1% Luria–Bertani (LB) agarose gels stained with ROTI^®^GelStain (Carl Roth, Karlsruhe, Germany) and sent to Microsynth Austria GmbH (Vienna, Austria) for purification and sequencing in both directions using the PCR primers. The forward and reverse sequences were aligned with BioEdit v. 7.0.5.3 [17], and the electropherograms were checked visually.

### Real-time quantitative PCRs

All qPCRs were run on a 7500 Fast Real-Time PCR System (Applied Biosystems, Waltham, MA, USA). The qPCRs with the TaqMan probes were performed using the Luna Universal Probe One-Step RT-qPCR Kit (New England Biolabs, Frankfurt, Germany). The TaqMan qPCRs for *P. relictum* SGS1 were conducted with the primers SGS1_Q160_F/SGS1_Q160_R and the probes SGS1-Pr1_Q and SGS1-Pr2_Q. The TaqMan qPCRs for *H. tartakovskyi* SISKIN1 were conducted with the primers SISKIN1_Q190_F/SISKIN1_Q190_R and the probes SISKIN1-Pr1_Q, SISKIN1-Pr2_Q, and SISKIN1-Pr3_Q. Three replicates were performed for each sample. The qPCRs were conducted in 20 µl volumes containing 10 µl Luna Universal Probe qPCR Mix (1×), 6 µl nuclease-free water, each 0.8 µl forward and reverse primer (10 µM), 0.4 µl probe (10 µM), and 2 µl template. The qPCRs were run under the following conditions: initial denaturation at 95 °C for 1 min followed by 40 cycles with denaturation phases at 95 °C for 15 s and extension phases at 60 °C for 30 s.

The dye-based qPCRs for *P. relictum* SGS1 were performed with the Luna^®^ Universal qPCR Master Mix (New England Biolabs) with the primers SGS1_Q1_F and SGS1_Q1_R specifically targeting the first 18S variant and SGS1_Q2_F and SGS1_Q2_R targeting the second 18S variant. The qPCRs were conducted in 20 µl volumes containing 10 µl Luna^®^ Universal qPCR Master Mix (1×), 7 µl nuclease-free water, each 0.5 µl forward and reverse primer (10 µM), and 2 µl cDNA as template. The dye-based qPCRs were run under the following conditions: initial denaturation at 95 °C for 1 min followed by 40 cycles with denaturation phases at 95 °C for 15 s and extension phases at 52 °C for 30 s.

### Histology and chromogenic in situ hybridization

To investigate differential 18S rRNA expression in tissue sections, organs of four donor birds (two birds each infected with SGS1 and SISKIN1) and whole dipteran vectors (12 SGS1-infected *Cx. quinquefasciatus*, 7 SISKIN1-infected *Cu. nubeculosus*, as well as uninfected controls) were subjected to histological investigation and CISH using the 18S rRNA variant-specific probes. The four siskins used as donor birds for the SGS1 and SISKIN1 experiments were euthanized by decapitation according to the permit obtained by the NRC in Vilnius (see Ethical statement). After dissection, organs (heart, lung, liver, spleen, kidney, brain, muscle, gizzard, intestine, trachea, and esophagus) were fixed in 10% neutral buffered formalin for 24 h, washed for 1 h in distilled water, and stored in 70% EtOH for shipping to the Vetmeduni. Mosquitoes and biting midges were treated the same way. The insects and bird organs were dehydrated through an increasing series of 70–100% ethanol, clarified in xylene, and individually embedded in paraffin wax. From each bird, 1–2 µm serial sections were cut from the organs and mounted on separate glass slides, one of which was stained with hematoxylin–eosin (HE), and the others were subjected to CISH. For HE-staining, regular glass slides were used, whereas sections were mounted on SuperFrost Plus Adhesion slides (Epredia, Fisher Scientific, Vienna, Austria) for CISH. For the analysis of vectors, serial sagittal sections of 1–2 µm were prepared from whole embedded dipterans until they were entirely cut, and all sections were mounted on SuperFrost Plus Adhesion slides. To identify the sections containing relevant body regions of the insects (head and thorax with salivary glands, and abdomen with midgut), every 10th section prepared was HE-stained for histological examination, while the remaining sections were reserved for CISH. For CISH, sections were deparaffinized, rehydrated in a series of graded ethanol (100%, 96%, 70%) and deionized water, and subjected to proteolytic treatment with proteinase K (#03115828001, Merck, Darmstadt, Germany) 3 μg/ml in tris-buffered saline for 40 min at 37 °C. Then, sections were rinsed in distilled water, dehydrated in 96% and 100% ethanol, air-dried, and incubated with a hybridization solution containing 10 ng digoxigenin-labeled probe per 100 µl. Of the SGS1-infected donor birds (*n* = 2), two serial sections each were separately hybridized with the probes SGS1-Pr1_ISH and SGS1-Pr2_ISH, while from the infected mosquitoes (*n* = 12) and uninfected controls (*n* = 3), each 8–12 sections were separately hybridized with the same probes. Similarly, of the SISKIN1-infected donor birds (*n* = 2), each three serial sections were separately hybridized with the probes SISKIN1-Pr1_ISH, SISKIN1-Pr2_ISH, and SISKIN1-Pr3_ISH, while from the infected biting midges (*n* = 7) and the uninfected controls (*n* = 2), each 10 sections were separately incubated with the three probes. As positive controls for testing the binding of the probes, each section of SGS1- and SISKIN1-infected birds was incubated with previously established genus-specific probes for *Plasmodium* (Plasmo18S) and *Haemoproteus* (Haemo18S), respectively [18]. Hybridizations were carried out overnight at 40 °C, placing the slides in a humid chamber. On the next day, sections were subjected to stringency washes in 2×, 1×, and 0.1× saline–sodium citrate (SSC) buffer for 10 min each before tissue sections were covered with a blocking solution containing normal goat serum and 10% Triton X-100 for 30 min. Thereafter, anti-digoxigenin-AP Fab-fragments (Roche, Basel, Switzerland) were applied at a concentration of 1:200 for 1 h at room temperature, followed by two washing steps with distilled water for 15 min. Then, tissue sections were incubated with the chromogenic substrates NBT/BCIP (nitro-blue tetrazolium chloride/5-bromo-4-chloro-3′-indolyphosphate *p*-toluidine salt, Roche) mixed with levamisole in 0.1 M tris-buffered saline (pH 9.5) for 40 min in a dark, humid chamber. After stopping the chromogenic reaction with tris–ethylenediaminetetraacetic acid (EDTA) buffer (pH 8.0) for 10 min, sections were counterstained with hematoxylin and mounted using Aquatex (Merck Millipore; Burlington, MA, USA) and coverslips.

## Results

### RNA extraction from bird blood and insect bodies

The total RNA extraction of *Cx. quinquefasciatus* body parts rendered between 90.5 ng and 1143.1 ng (mean 583.2 ng) cDNA for the abdomens and between 56.1 ng and 965.1 ng (mean 441.7 ng) of cDNA for the head/thorax, while for the bird blood samples between 210.4 ng and 452.2 ng (mean 347.3 ng) cDNA were obtained. The *Cu. nubeculosus* body parts rendered between 62.3 ng and 779.9 ng (mean 272.0 ng) cDNA for the abdomens and between 56.4 ng and 404.1 ng (mean 112.9 ng) for the head/thorax, and the birds’ blood between 99.6 ng and 409.8 ng (mean 299.3 ng) cDNA.

### PCRs for parasite detection in cDNA samples and real-time quantitative PCRs

#### Plasmodium relictum SGS1

**Fig. 2:**
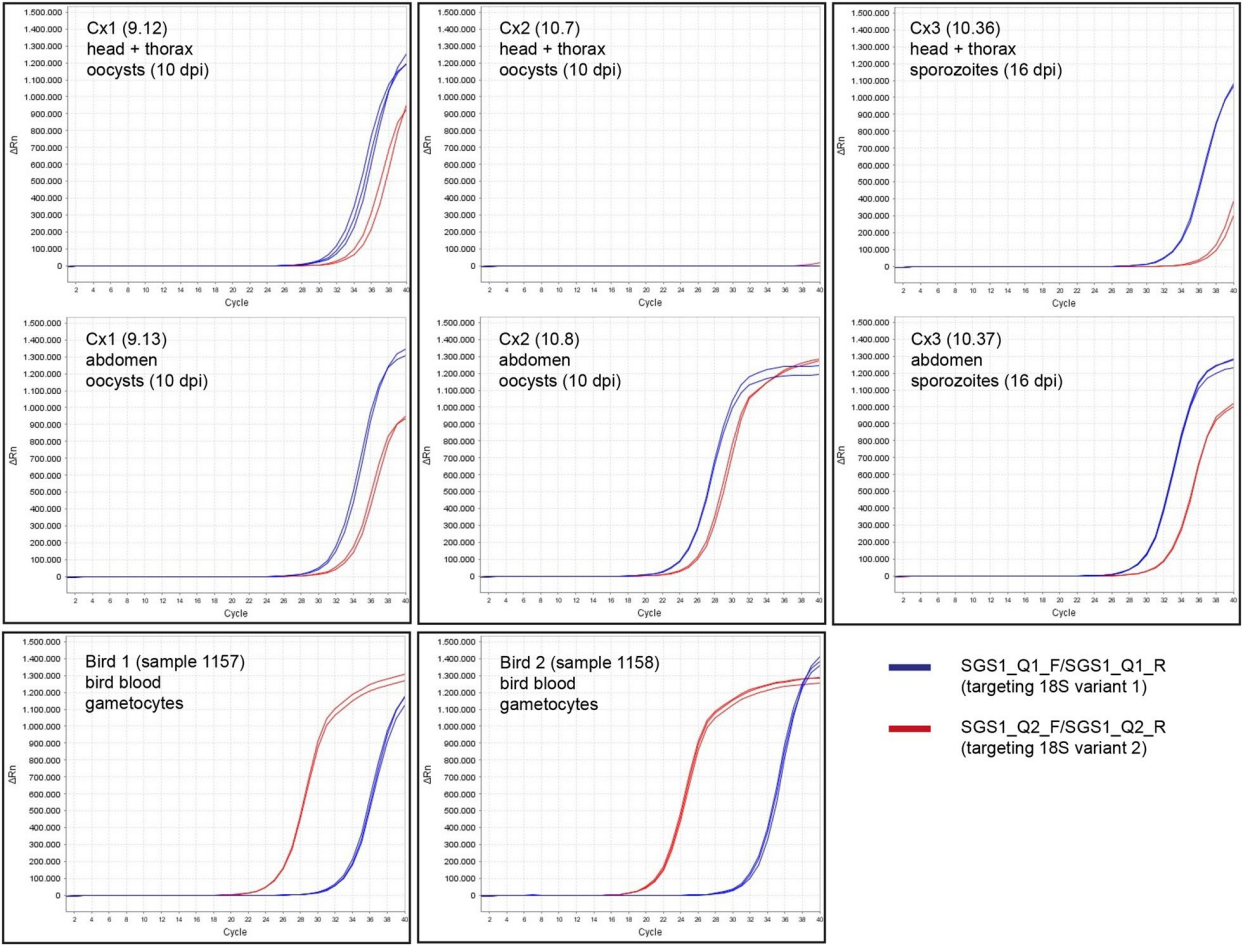
Real-time quantitative PCRs (dye-based) targeting the 18S rRNA of *Plasmodium relictum* SGS1 in *Culex quinquefasciatus* mosquitoes and bird blood with variant-specific primers

The qPCRs for the *P. relictum* SGS1 experiment included 12 *Cx. quinquefasciatus* mosquitoes sampled at 9–10 dpi, 14 mosquitoes sampled at 16–17 dpi, and one uninfected mosquito as negative control. Parasite rRNA was detected by standard PCR in cDNA samples of two mosquitoes (16.7%) sampled at 9–10 dpi and one mosquito (7.1%) sampled at 16–17 dpi. In one of the two individuals sampled at 10 dpi (individual Cx2; oocyst stage), the abdomen was positive, but the head/thorax was negative. In the second individual (Cx1), both abdomen and head/thorax were positive. In the mosquito sampled at 16 dpi (Cx3; sporozoite stage), both abdomen and head/thorax were positive. The blood samples taken from the two donor birds (Bird 1: 1157; Bird 2: 1158) were all PCR-positive. Direct sequencing of PCR products showed that 18S variant 1 (targeted by probe SGS1-Pr1_Q in the TaqMan qPCRs) was dominating in the mosquitoes, while 18S variant 2 (targeted by probe SGS1-Pr2_Q) was dominating in the bird blood. However, in the TaqMan qPCR assay, only the expression of 18S variant 2 (targeted by probe SGS1-Pr2_Q) could be detected, but no signals were detected for the expression of 18S variant 1 (targeted by probe SGS1-Pr1_Q) (Additional file 1: Fig. S1). Due to the assumed failure of probe SGS1-Pr1_Q in the TaqMan qPCR assay, a dye-based qPCR approach with the variant-specific primer sets SGS1_Q1_F/SGS1_Q1_R and SGS1_Q2_F/SGS1_Q2_R was performed in addition. The dye-based qPCR approach allowed the detection of both variants in the mosquitoes. Although the expression levels of the two variants were generally similar in the mosquitoes, the results showed lower CT values for 18S variant 1, indicating higher expression. By contrast, in the bird blood samples, 18S variant 2 was considerably more strongly expressed than 18S variant 1 (Table 3, Fig. 2).

**Table 3:** CT values (repeats, mean, and standard deviation) resulting from the dye-based real-time quantitative PCRs targeting the two 18S variants of *Plasmodium relictum* SGS1 in *Culex quinquefasciatus* mosquitoes and bird blood with variant-specific primers

IDs (mosquitoes, birds)	dpi	Body part	SGS1_Q1_F/SGS1_Q1_R (variant 1)					SGS1_Q2_F/SGS1_Q2_R (variant 2)				
			rep 1	rep 2	rep3	CT mean	CT SD	rep 1	rep 2	rep3	CT mean	CT SD
Cx1 (9.13)	10	Abdomen	24.19	23.79	24.44	24.14	0.33	26.95	26.61	25.74	26.43	0.62
(9.12)	10	Head/thorax	24.27	25.31	24.91	24.83	0.52	28.26	28.80	27.59	28.22	0.61
Cx2 (10.8)	10	Abdomen	17.46	17.24	17.05	17.25	0.21	19.34	19.65	19.07	19.35	0.29
(10.7)	10	Head/thorax	−	−	−	−	−	−	−	−	−	−
Cx3 (10.37)	16	Abdomen	20.52	23.42	22.96	22.30	1.56	26.30	25.94	26.08	26.11	0.18
(10.36)	16	Head/thorax	26.23	26.14	26.65	26.34	0.27	31.57	31.61	30.55	31.24	0.60
Bird 1 (1157)	−	Blood	25.98	25.68	26.25	25.97	0.29	19.38	19.60	18.95	19.31	0.33
Bird 2 (1158)	−	Blood	24.95	22.92	25.87	24.58	1.51	15.74	15.59	15.64	15.66	0.08

“−” negative: no parasite 18S rRNA detected

#### *Haemoproteus tartakovskyi* SISKIN1

**Fig. 3:**
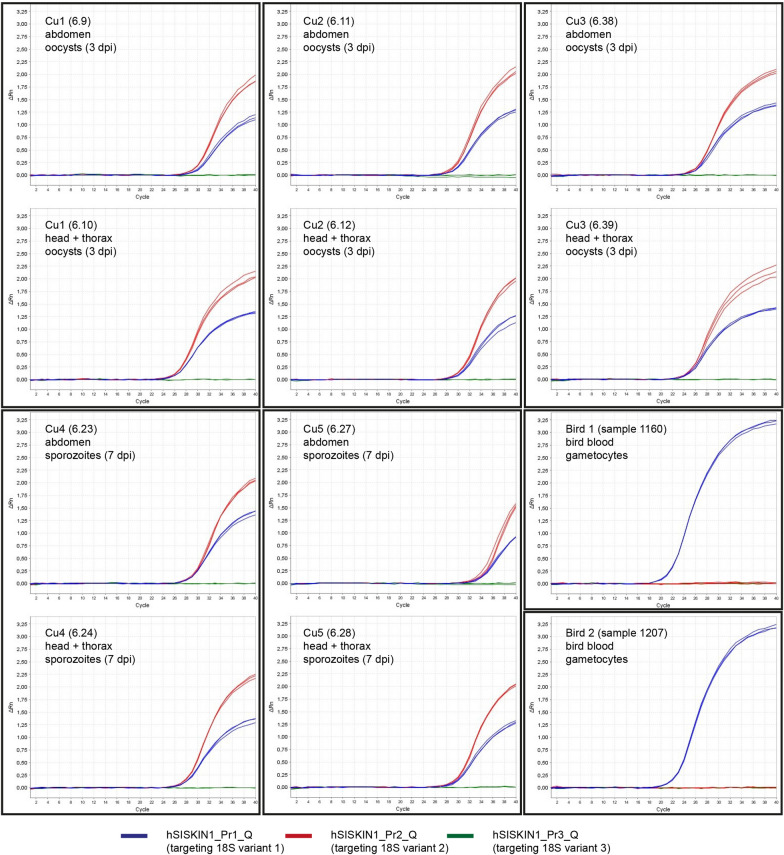
Real-time quantitative PCRs (TaqMan assay) targeting the 18S rRNA of *Haemoproteus tartakovskyi* SISKIN1 in *Culicoides nubeculosus* biting midges and bird blood with variant-specific probes (first plate)

**Fig. 4:**
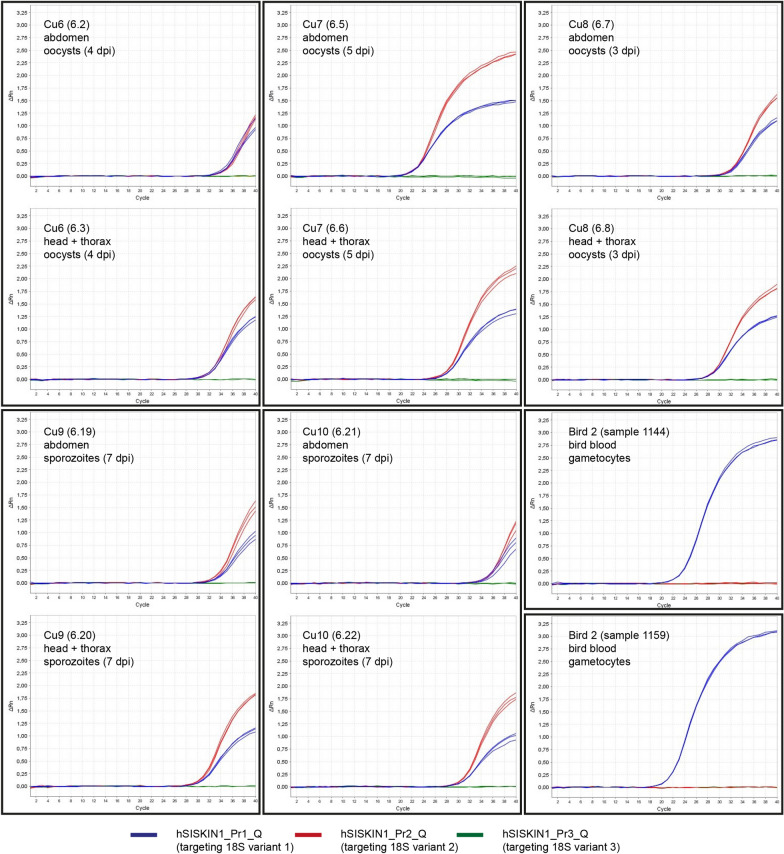
Real-time quantitative PCRs (TaqMan assay) targeting the 18S rRNA of *Haemoproteus tartakovskyi* SISKIN1 in *Culicoides nubeculosus* biting midges and bird blood with variant-specific probes (second plate)

The qPCRs for the *H. tartakovskyi* SISKIN1 experiment included seven *Cu. nubeculosus* biting midges sampled at 3–5 dpi (oocyst stage), six individuals sampled at 7 dpi (sporozoite stage), and two uninfected controls. Standard PCRs conducted on cDNA samples with the primers SISKIN1_Q190_F and SISKIN1_Q190_R detected 18S rRNA in six individuals sampled at 3–5 dpi (85.7%) and four individuals sampled at 7 dpi (66.7%). In the case of the six PCR-positive individuals sampled at 3–5 dpi, both body parts were positive. Strong PCR signals were detected in all PCR-positive head/thorax samples of the individuals sampled at 7 dpi, whereas the abdomen samples provided weak signals in three cases. The blood samples of the two donor birds (Bird 1: 1144, 1159, 1207; Bird 2: 1160) were all PCR-positive. Direct sequencing of PCR products showed that in the bird blood, only the 18S variant targeted by probe hSISKIN-Pr1_Q was expressed. By contrast, in the biting midges, 18S variant 2, targeted by the probe hSISKIN-Pr2_Q, was dominating, but double peaks in the electropherograms indicated that 18S variant 1 was expressed in parallel. This was confirmed by the qPCR results, which also revealed higher expression levels for variant 1 compared to variant 2. There was no obvious difference depending on the assumed developmental stage (oocysts or sporozoites). By contrast, in the bird blood, only 18S variant 1 (targeted by probe hSISKIN-Pr1_Q) was expressed according to direct sequencing and qPCR results, thus supporting differential expression of the 18S rRNA genes in bird hosts and biting midges (Table 4, Figs. 3, 4). Surprisingly, the third 18S variant (SISKIN1-Pr3_Q) was not expressed either by oocysts or sporozoites in the biting midges or by gametocytes in the bird blood.

**Table 4:** CT values (repeats, mean, and standard deviation) resulting from the real-time quantitative PCRs (TaqMan assay) targeting the three 18S variants of *Haemoproteus tartakovskyi* SISKIN1 in *Culicoides nubeculosus* and bird blood of *Spinus spinus* with variant-specific probes

IDs (biting midges, birds)	dpi	Body part	SISKIN1-Pr1_Q (variant 1)					SISKIN1-Pr2_Q (variant 2)				
			rep 1	rep 2	rep 3	CT mean	CT SD	rep 1	rep 2	rep 3	CT mean	CT SD
Cu1 (6.9)	3	Abdomen	31.35	31.26	30.99	31.20	0.19	30.37	30.43	30.52	30.44	0.08
(6.10)	3	Head/thorax	27.69	27.73	27.66	27.69	0.04	27.22	27.23	27.33	27.26	0.06
Cu 2 (6.11)	3	Abdomen	30.74	30.55	30.50	30.60	0.13	29.89	30.09	30.03	30.00	0.10
(6.12)	3	Head/thorax	31.65	31.32	31.51	31.49	0.17	31.07	31.00	30.95	31.01	0.06
Cu3 (6.38)	4	Abdomen	26.77	26.49	26.53	26.60	0.15	26.84	26.74	26.85	26.81	0.06
(6.39)	4	Head/thorax	24.89	25.28	25.28	25.15	0.23	25.45	25.46	25.73	25.55	0.16
Cu4 (6.23)	7	Abdomen	28.70	28.73	28.79	28.74	0.05	28.26	28.18	27.94	28.13	0.17
(6.24)	7	Head/thorax	27.87	27.80	27.97	27.88	0.09	26.99	26.96	26.99	26.98	0.02
Cu5 (6.27)	7	Abdomen	34.65	34.91	34.84	34.80	0.13	35.09	34.93	34.43	34.82	0.34
(6.28)	7	Head/thorax	30.60	30.55	30.33	30.49	0.14	30.68	30.53	30.49	30.57	0.10
Cu6 (6.2)	4	Abdomen	34.88	34.90	34.49	34.76	0.23	35.46	35.72	35.88	35.69	0.21
(6.3)	4	Head/thorax	32.01	32.41	32.44	32.29	0.24	33.04	32.74	32.73	32.84	0.18
Cu7 (6.6)	5	Abdomen	28.37	28.28	28.40	28.35	0.06	28.47	28.51	28.59	28.52	0.06
(6.5)	5	Head/thorax	22.69	22.78	22.74	22.74	0.05	23.13	23.16	23.29	23.19	0.09
Cu8 (6.7)	3	Abdomen	33.46	33.15	33.24	33.28	0.16	33.10	32.96	33.04	33.03	0.07
(6.8)	3	Head/thorax	29.96	29.85	29.99	29.93	0.07	29.74	29.76	29.74	29.75	0.01
Cu9 (6.19)	7	Abdomen	33.48	33.56	33.79	33.61	0.16	32.31	32.88	32.48	32.56	0.29
(6.20)	7	Head/thorax	30.66	30.82	30.80	30.76	0.09	29.90	29.71	29.65	29.75	0.13
Cu10 (6.21)	7	Abdomen	35.58	34.74	35.03	35.12	0.43	34.42	34.46	34.73	34.54	0.17
(6.22)	7	Head/thorax	30.87	30.84	30.86	30.86	0.02	29.90	29.87	29.89	29.89	0.02
Bird 1 (XT07053; 1160)	0	Blood	21.20	21.24	21.30	21.25	0.05	−	−	−	−	−
Bird 2 (XT08772; 1144)	0	Blood	22.48	22.54	22.50	22.51	0.03	−	−	−	−	−
Bird 2 (XT08772; 1159)	0	Blood	21.34	21.31	21.37	21.34	0.03	−	−	−	−	−
Bird 2 (XT08772; 1207)	0	Blood	22.28	22.42	22.34	22.35	0.07	−	−	−	−	−

Results for the third probe are not shown because the respective qPCRs were all negative

“−” Negative: no parasite 18S rRNA detected

### Chromogenic in situ hybridization

#### Plasmodium relictum SGS1

**Fig. 5:**
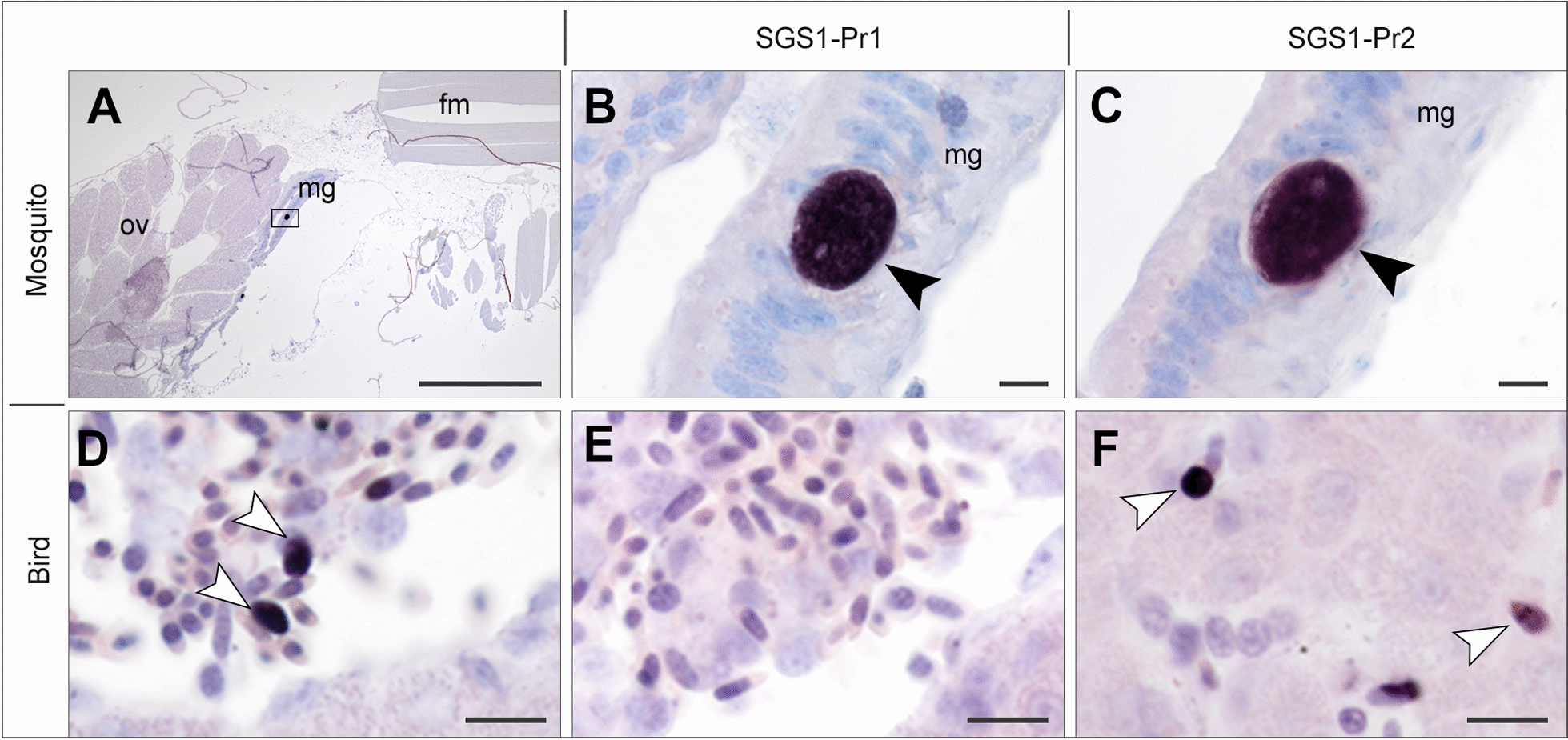
Chromogenic in situ hybridization targeting two 18S variants of *Plasmodium relictum* SGS1 in experimentally infected *Culex quinquefasciatus* (**A**–**C**) and Eurasian siskins (**D**–**F**). In *Cx. quinquefasciatus* (Cx11, 10 dpi), oocysts (black arrowheads) in the midgut were stained by both probes (**B**, **C**). In the Eurasian siskin (Bird 2), SGS1 blood stages (white arrowheads) were visualized in blood vessels of the liver using a *Plasmodium* genus-specific probe (Plasmo18S) (**D**). Applying the 18S variant-specific probes showed labeling of gametocytes with SGS1-Pr2 (**F**), but not with SGS1-Pr1 (**E**). Ov: ovary; mg: midgut; fm: flight muscle. Scale bars: 500 µm (**A**) and 10 µm (**B**–**F**)

CISH applying the two *P. relictum* 18S variant-specific probes to sections of 12 infected mosquitoes revealed parasites in only two individuals sampled at 10 dpi (Cx4 and Cx11) (Table 5). In these, several oocysts were detected in the midgut epithelium and labeled by both 18S variant-specific ISH probes, SGS1-Pr1 and SGS1-Pr2 (Fig. 5A–C). Interestingly, some oocysts present in the midgut epithelium remained unstained (Additional file 2: Fig. S2). Despite careful examination of the salivary glands, stained or unstained sporozoites were not observed in any of the four mosquitoes collected at 16 dpi, suggesting a failure of infection in these individuals. All three uninfected control mosquitoes were negative by CISH. Using the *Plasmodium* genus-specific probe, CISH testing of one SGS1-infected donor bird showed multiple small, roundish signals in capillaries and vessels of various tissues, corresponding to blood stages of the parasites (Fig. 5D). Applying the two 18S variant-specific ISH probes showed staining of parasite blood stages with the probe SGS1-Pr2 (Fig. 5F) but not with the probe SGS1-Pr1 (Fig. 5E). This generally corresponded to the results of the PCR assays, although in the dye-based qPCR assays the second 18S variant was expressed in bird blood as well but at lower levels than the first variant.

**Table 5:** Results of CISH applying 18S variant-specific probes to tissue sections of *Culex quinquefasciatus* infected with *Plasmodium relictum* SGS1

ID^1^	Dpi	Developmental stage	*N* sections tested per probe	Parasite stages labeled by CISH^2,3^	
				SGS1-Pr1_ISH	SGS1-Pr2_ISH
Cx4 (9.8)	10 dpi	Oocyst	12	Oocysts (mg)	Oocysts (mg)
Cx5 (9.9)	10 dpi	Oocyst	12	−	−
Cx6 (9.14)	10 dpi	Oocyst	8	−	−
Cx7 (10.5)	10 dpi	Oocyst	8	−	−
Cx8 (9.14)	10 dpi	Oocyst	8	−	−
Cx9 (10.5)	10 dpi	Oocyst	8	−	−
Cx10 (10.6)	10 dpi	Oocyst	8	−	−
Cx11 (10.11)	10 dpi	Oocyst	8	Oocysts (mg)	Oocysts (mg)
Cx12 (10.26)	16 dpi	Sporozoite	12	−	−
Cx13 (10.27)	16 dpi	Sporozoite	12	−	−
Cx14 (10.28)	16 dpi	Sporozoite	8	−	−
Cx15 (10.29)	16 dpi	Sporozoite	8	−	−
Cx16 (10.39)	16 dpi	Sporozoite	8	−	−
Cx17 (10.40)	16 dpi	Sporozoite	8	−	−

^1^Uninfected control individuals were not included in the table

^2^“−” Negative (no labeled parasite stages seen)

^3^Location of detected parasite stages is indicated in brackets: mg, midgut

#### *Haemoproteus tartakovskyi* SISKIN1

**Fig. 6:**
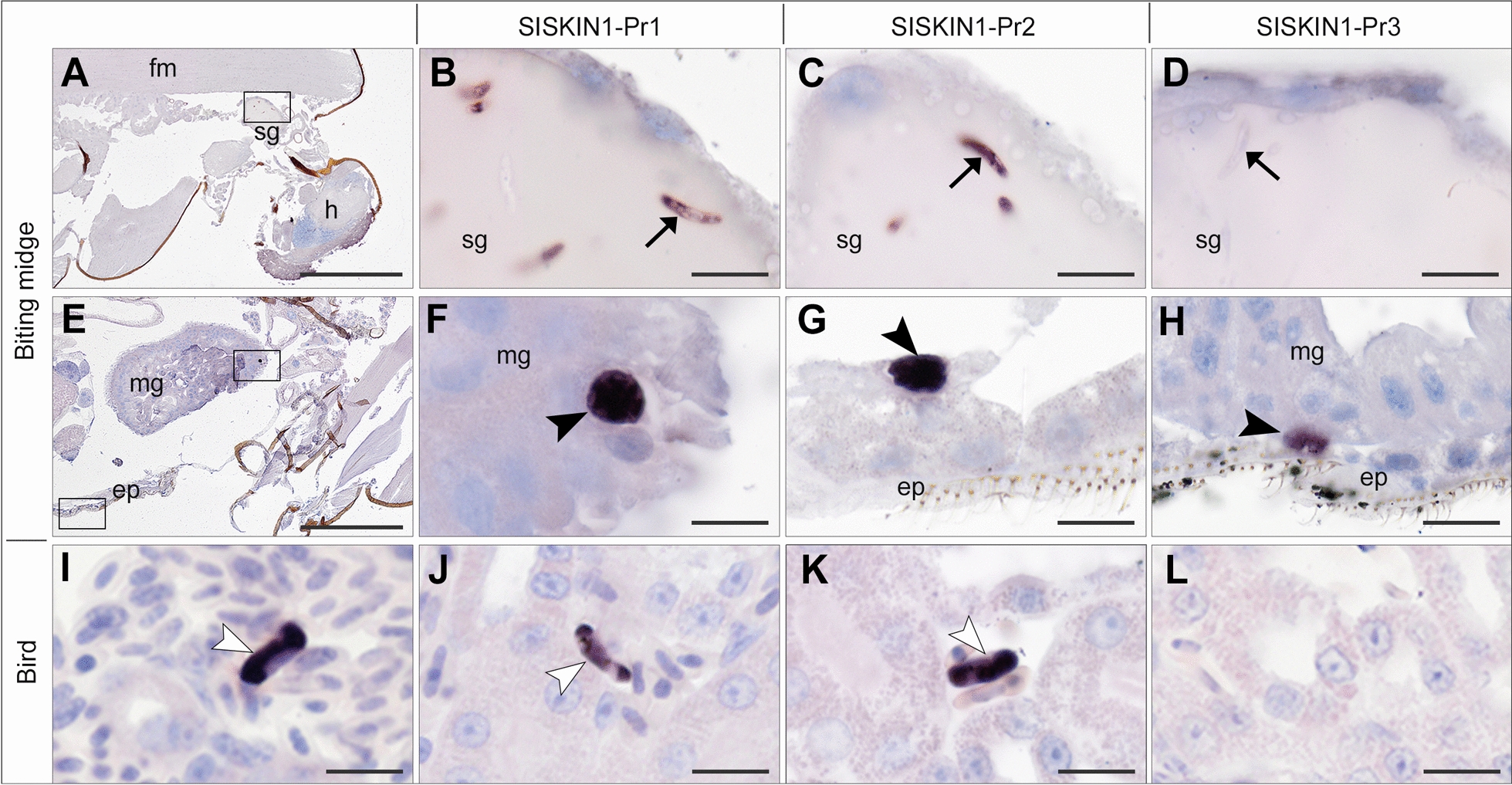
Chromogenic in situ hybridization assay targeting three 18S variants of *Haemoproteus tartakovskyi* SISKIN1 in experimentally infected *Culicoides nubeculosus* (**A**–**H**) and Eurasian siskins (**I**–**L**). In *Cu. nubeculosus* (Cu11, 3 dpi), sporozoites (arrows) in salivary glands were labeled by the ISH probes SISKIN1-Pr1 (**B**) and SISKIN-Pr2 (**C**) but not by SISKIN-Pr3 (**D**), whereas oocysts were labeled by all three probes (**F**–**H**). In the Eurasian siskin, parasite blood stages (white arrowheads) were visualized in blood vessels using a *Haemoproteus* genus-specific probe (Haemo18S) (**I**). Applying the 18S variant-specific probes showed labeling of gametocytes in kidney capillaries with SISKIN1-Pr1 (**J**) and SISKIN1-Pr2 (**K**) but not with SGS1-Pr3 (**L**). Sg: salivary gland; h: head; fm: flight muscle; mg: midgut; ep: epidermis. Scale bars: 200 µm (**A**, **E**) and 10 µm (**B**–**D**, **F**–**L**)

CISH applying the three *H. tartakovskyi* 18S variant-specific probes to sections of seven infected biting midges revealed parasite stages in four individuals sampled at 3 dpi (Table 6). In all of them, sporozoites were detected in the salivary glands, while oocysts were only found in two individuals. Both sporozoites (Fig. 6B–D) and oocysts (Fig. 6F–H) were labeled by the two probes targeting variant 1 (SISKIN1-Pr1) and 2 (SISKIN1-Pr2). Interestingly, the probe targeting variant 3 (SISKIN1-Pr3) also showed staining of oocysts in two individuals (Fig. 6H), while sporozoites present in salivary glands remained unstained (Fig. 6D). The labeling of oocysts with this probe was surprising because the corresponding TaqMan probe SISKIN1-Pr3_Q did not produce signals in the qPCRs. Interestingly, labeled oocysts were not only found in the midgut epithelium but also located within or in close association to the epidermis of the ventral abdomen (Fig. 6G, H; Additional file 3: Fig. S3). All uninfected control biting midges were negative by CISH with the three probes.

**Table 6:** Results of CISH applying 18S variant-specific probes on tissue sections of *Culicoides nubeculosus* infected with *Haemoproteus tartakovskyi* SISKIN1

ID^1^	Dpi	Developmental stage^1^	*N* sections tested per probe	Parasite stages located and labeled by CISH^2,3^		
				SISKIN1-Pr1_ISH	SISKIN1-Pr2_ISH	SISKIN1-Pr3_ISH
Cu11 (6.16)	3 dpi	Oocyst	10	Sporozoites (sg), oocysts (mg, epi)	Sporozoites (sg, mg), oocyst (epi)	oocyst (epi)
Cu12 (6.17)	3 dpi	Oocyst	10	Sporozoites (sg), oocysts (epi)	Sporozoites (sg), oocysts (epi)	oocyst (epi)
Cu13 (6.18)	3 dpi	Oocyst	10	Sporozoites (sg)	Sporozoites (sg)	−
Cu14 (6.15)	3 dpi	Oocyst	10	Sporozoites (sg)	Sporozoites (sg)	−
Cu15 (6.4)	4 dpi	Oocyst	10	−	−	−
Cu16 (6.33)	7 dpi	Sporozoite	10	−	−	−
Cu17 (6.34)	7 dpi	Sporozoite	10	−	−	−

^1^Uninfected control individuals were excluded from the table

^2^ “−” Negative (no labeled parasite stages seen)

^3^Location of detected parasite stages is indicated in brackets: mg, midgut; sg, salivary gland; epi, epidermis

CISH testing of the *H. tartakovskyi* SISKIN1-infected donor birds using the *Haemoproteus* genus-specific probe showed labeling of parasite blood stages in various tissues of both birds (Fig. 6I), confirming the presence of gametocytes. Applying the three 18S variant-specific ISH probes on the same birds’ tissues showed labeling of blood stages with the probes targeting variants 1 and 2 (SISKIN-Pr1, SISKIN-Pr2) (Fig. 6J, K), but not with the probe targeting variant 3 (SISKIN1-Pr3) (Fig. 6L). Staining of gametocytes with the probe SISKIN1-Pr2_ISH was unexpected because the corresponding TaqMan probe SISKIN1-Pr2_Q did not produce signals in the qPCRs performed on cDNA samples from bird blood.

## Discussion

This study aimed to investigate the differential expression of distinct 18S rRNA gene variants in bird hosts and dipteran vectors of the haemosporidian parasite species *P. relictum* SGS1 and *H. tartakovskyi* SISKIN1. The question was addressed using two different approaches. In the first approach, the expression of the 18S rRNA variants was assessed using qPCRs with cDNA obtained from RNA extracts of body parts of the dipteran vectors and bird blood. In the second approach, the parasites were targeted by CISH in tissue sections of whole dipteran vectors and bird organs. The results of the study confirmed our hypothesis on differential 18S rRNA gene expression in the bird hosts and vectors, and, for the first time, provided visualizations of avian haemosporidian parasite stages in tissue sections other than midgut of the vectors, with the discovery of extra-midgut development of oocysts in the epidermis of the ventral abdomen of *H. tartakovskyi* SISKIN1-infected biting midges.

### Differential expression of 18S rRNA genes in *Plasmodium relictum* SGS1

The dye-based qPCRs performed for the *P. relictum* SGS1 experiment showed that both 18S variants were expressed in mosquitoes and birds but at different levels. In the mosquitoes, variant 1 was slightly more strongly expressed than variant 2, whereas in the birds, the opposite was the case, with an even more prominent difference between the two variants. This was also evident when directly sequencing the PCR products obtained with primers targeting both variants in cDNA samples, showing that variant 1 dominated in the mosquitoes and variant 2 in the birds. These results were confirmed by CISH using the variant-specific probes, in which both probes (targeting variants 1 and 2) stained oocysts in the mosquitoes. This contrasts slightly with the qPCR results indicating lower expression of variant 2, thus, less intense CISH staining with the probe targeting variant 2 was expected. However, considering the small differences between the expression levels of variants 1 and 2 in the mosquitoes, this might not be reflected in CISH staining intensities as the overall expression was strong for both 18S variants. The experiments confirmed our hypothesis on the differential expression of two distinct 18S variants of *P. relictum* SGS1 in mosquitoes and birds. However, the experimental infections of *Cx. quinquefasciatus* mosquitoes with *P. relictum* SGS1 were suboptimal since parasites were detected by PCR in only two of eight mosquitoes sampled at 9–10 dpi and only one of eight mosquitoes sampled at 16–17 dpi. Similarly, oocysts were detected by CISH in the midgut of only two of the 12 mosquitoes sampled at 9–10 dpi, while no oocysts were found in the 12 mosquitoes sampled at 16 dpi. Thus, including a larger number of mosquitoes in the experiment would have been recommended to obtain more robust results. The low infection rates might be related to the choice of the vector species. *Culex quinquefasciatus* is distributed in subtropical and tropical areas and is the main vector of the genetically distinct lineage *P. relictum* GRW04 [19]. Since *P. relictum* SGS1 is mainly transmitted by *Cx. pipiens* mosquitoes in temperate regions [20], *Cx. quinquefasciatus* might be a suboptimal vector for *P. relictum* SGS1. In comparison, Kazlauskienė et al*.* [21] experimentally infected wild-caught *Cx. pipiens* f. *pipiens* mosquitoes with *P. relictum* SGS1 strains from canaries and crossbills and found parasite stages in all dissected individuals (ookinetes between 1 and 6 dpi, oocysts between 6 and 24 dpi, and sporozoites between 14 and 32 dpi).

Sekar et al. [22] published the only study in which they successfully infected *Cx. quinquefasciatus* mosquitoes with *P. relictum* SGS1 but did not indicate the success rate. They observed oocysts in the mosquito midguts as soon as 4–6 dpi (peak: 8–10 dpi) and sporozoites in the head and thorax at 8–10 dpi (peak: 10 dpi) [22]. Using the natural vector *Cx. pipiens pipiens* likely would have resulted in higher infection rates and more robust results but would have required collecting hibernating mosquitoes as done by Kazlauskienė et al*.* [21]. However, it would have been challenging to collect sufficient numbers of wild mosquitoes, and they potentially could have been naturally infected with *Plasmodium* parasites. It is also possible that the cryopreserved *P. relictum* SGS1 strain had a decreased ability to infect the vectors because it was passaged at least three times in birds before. Such a phenomenon was observed in *Plasmodium lophurae*, a parasite of Phasianidae birds, which lost the ability to produce gametocytes after numerous blood passages in laboratory hosts [23, 24].

### Differential expression of 18S rRNA genes in *Haemoproteus tartakovskyi* SISKIN1

The qPCRs performed for the *H. tartakovskyi* SISKIN1 experiment showed that only one of the three 18S variants (variant 1) was expressed in the birds, while two variants (variant 1 and 2) were simultaneously expressed in the biting midges. In contrast, expression of the third 18S variant was not detected in either birds or biting midges by qPCR. This was also supported by directly sequencing the PCR products obtained with primers targeting all three variants in the cDNA samples obtained after RNA extraction. The results were generally confirmed by CISH using the variant-specific probes, but surprisingly, the probe targeting 18S variant 3 also stained oocysts in the biting midges, providing indirect evidence of expression of the third 18S variant in these parasite stages. Moreover, the same probe selectively stained oocysts but not sporozoites present in salivary glands of the biting midges (Fig. 6D). Given the possibility that the third 18S variant might be primarily expressed by zygotes or ookinetes, parasite stages which are usually observed during the first dpi [25], and assuming a gradual switch from the expression of one 18S variant to another during ookinete-to-oocyst transformation, this could explain the labeling of oocysts with the probe targeting variant 3. It remains unclear why the qPCRs targeting variant 3 did not provide signals, but it has to be noted that different individuals were analyzed by qPCR and CISH, limiting direct comparisons between the data and interpretation thereof. So far, additional rRNA classes have only been found in two mammalian malaria parasites. A so-called O-type rRNA unit is expressed by ookinetes and oocysts of the human malaria parasite *P. vivax* [5], and the D-type rRNA unit is required for the normal development of oocysts and sporozoites in the rodent malaria parasite *P. yoelii* [6]. Additional studies are required to test whether the third 18S variant of *H. tartakovskyi* SISKIN1 is expressed in zygotes and ookinetes. Unfortunately, for the present study, we did not collect biting midges during the first 2 dpi for RNA extraction and CISH, which supposedly would have contained these parasite stages.

The infection of biting midges was successful in most cases. Parasites were detected by PCR in six of seven biting midges sampled at 3–5 dpi and four of six biting midges sampled at 7 dpi. Using CISH, parasites were detected in four of five biting midges sampled at 3–4 dpi but in none of the two individuals sampled at 7 dpi, which were assumed to contain both oocysts and sporozoites. Surprisingly, sporozoites were detected in two biting midges sampled at 3 dpi although this parasite stage was not expected to appear that early. In a previous study, *Cu. nubeculosus* biting midges had been experimentally infected with *H. tartakovskyi* SISKIN1 under lower temperatures (22–23 °C compared to 27 °C in the present study) and zygotes and ookinetes formed from 36 h post-infection (hpi) to 2 dpi, oocysts at 3–4 dpi, and sporozoites at 7–11 dpi [25]. Similarly, the rapid development of sporozoites as in the present study was observed for *Leucocytozoon* (*Akiba*) *caulleryi*, which developed sporozoites at 2–3 dpi after exposure at 25 °C [26, 27].

Another interesting observation was the location of oocysts in or close to the ventral epidermis. Generally, oocysts of *Haemoproteus* parasites develop in the midgut epithelium, but previous studies have shown that ookinetes, which possess a conoid [28, 29], also traverse the midgut wall and can be found in the haemocoel all over the body of insects [16]. Thus, it is conceivable that in the biting midges investigated in this study, ookinetes penetrated through the midgut wall and found their way to infect epidermal cells, which might have been facilitated by the distention of the midgut after blood meal and thus close alignment of midgut wall and epidermis. This observation is novel in avian haemosporidian parasites and provides the first evidence for extraintestinal development of *Haemoproteus* oocysts. CISH staining indicates that these oocysts express rRNA and therefore are viable. However, whether they produce sporozoites or represent a developmental dead-end remains to be investigated.

### Recent advances and prospects in transcriptomics of avian haemosporidian parasites

Since haemosporidian parasites feature numerous developmental stages in two strongly contrasting environments, vertebrate hosts and dipteran vectors, they are highly interesting organisms for studying differential gene expression. However, sequencing genomes and transcriptomes of avian haemosporidian parasites from bird blood is challenging, particularly because the amount of host DNA and RNA vastly exceeds that of the parasites: erythrocytes of birds are nucleated and contain considerably larger genomes (> 1200 Mb) than haemosporidian parasites (~ 23 Mb); genomes of red blood cells are diploid while parasite stages in the blood are haploid, parasitemia is often low, with less than 1% of blood cells being infected; and the high AT content in haemosporidian genomes results in fewer sequencing reads [30].

Differential gene expression not only affects rRNA genes but also affects numerous coding genes, which has been shown previously for mammalian *Plasmodium* species using proteomics and transcriptomics [31–34]. Avian *Plasmodium* parasites have been investigated using transcriptomics as well, studying whether the expression of genes differed between individuals of the same bird host [35], between different bird hosts [36], and between intermediate and peak infections [35, 37]. Sekar et al*.* [22] first studied differential gene expression of an avian *Plasmodium* species in birds and vectors by sequencing the transcriptomes of *P. relictum* SGS1 parasites in bird blood and during different developmental stages (30 min post-infection, 8 dpi, 12 dpi, and 22 dpi) in *Cx. quinquefasciatus* mosquitoes. Unfortunately, the latter study did not investigate the expression of the 18S genes, because rRNA was removed before cDNA library preparation and sequencing [22]. It would be important to conduct transcriptome studies on further avian *Plasmodium* species and parasites belonging to the genera *Haemoproteus* and *Leucocytozoon*, which constitute a large fraction of haemosporidian parasites found in bird hosts. As a basis for transcriptomic studies of non-*Plasmodium* species, it would be important to sequence the genomes of selected *Haemoproteus* and *Leucocytozoon* species, but so far only the genome of *H. tartakovskyi* SISKIN1 has been published [10].

Until now, the reasons for the maintenance of distinct 18S rRNA gene copies in haemosporidian parasites have not been sufficiently explained. Studies on *P. falciparum* showed that important factors triggering the expression of distinct 18S variants are varying temperature and glucose concentrations in the vertebrate host and mosquito vector [3, 38]. Van Spaendonk et al*.* [39] concluded that the structural differences between rRNA molecules of *P. berghei* do not support functional differences, because core regions of all rRNA molecules are similar, temporal differences in expression of the S-type rRNA gene units are lacking, knockout experiments showed that one S-type copy is sufficient for development, and both A-type and S-type variants are active in the mosquito host at the same time [39]. By contrast, McGee et al*.* [40] suggest that distinct rRNA types in *P. falciparum* and other malaria species could be of functional importance and that expansion segments (ES) interrupting the universal core secondary structure of rRNAs may have different structures and abilities to recruit specific effector proteins in vertebrate hosts and mosquito vectors [40].

It is important to mention that the divergence of intraspecific 18S variants varies strongly between *Plasmodium* species, ranging from 0.3% in *P. malariae* to 17.6% in *P. ovale wallikeri.* Similarly, distances between 18S variants of avian *Plasmodium* species ranged from 0.6% in *P. delichoni* to 14.9% in *P. elongatum* [8]. In contrast, most *Haemoproteus* and *Leucocytozoon* species investigated feature only slightly different 18S variants, except for *H. tartakovskyi* and species of the *L. toddi* group [9, 18]. Thus, it can be concluded that the possession of strongly diverged rRNA genes is not a universal feature within the genus *Plasmodium* and other haemosporidian parasites. Moreover, apicomplexan parasites of other orders have complex life cycles in vertebrate hosts and arthropod vectors as well, but they do not feature considerably distinct copies of the rRNA genes. Nonetheless, it can be assumed that strongly diverged rRNA gene variants provide an evolutionary benefit for those haemosporidian parasites possessing them.

## Conclusions

In this study, we show for the first time that the avian haemosporidian parasites *P. relictum* SGS1 and *H. tartakovskyi* SISKIN1 differentially express distinct 18S rRNA gene variants in bird hosts and dipteran vectors. For *P. relictum*, the qPCRs showed that both 18S variants were expressed in parallel in birds and mosquitoes, where one variant was more strongly expressed in the mosquitoes and the other in the birds. For *H. tartakovskyi*, featuring three distinct 18S variants, the qPCRs showed that only the first 18S variant was expressed in birds while the first and second 18S variants were both expressed in the biting midges, but no signals were detected for the third variant. These findings correspond with data on mammalian *Plasmodium* species, indicating that differential expression of ribosomal genes occurs in various groups of mammalian and avian haemosporidian parasites and their dipteran vectors (*Anopheles* and *Culex* mosquitoes and *Culicoides* biting midges). This might provide opportunities to explore gene expression of other avian haemosporidian parasites for which experimental setups involving vertebrate hosts and dipteran vectors have already been established. In addition, the CISH analyses provided the first visualizations of avian haemosporidian oocysts in tissue sections of the vectors, with the discovery of extraintestinal development of oocysts in *H. tartakovskyi* SISKIN1-infected biting midges.

## Supplementary Information

Additional file 1: Fig. S1. Real-time quantitative PCRs (TaqMan qPCR assay) targeting the 18S rRNA of *Plasmodium relictum* SGS1 in *Culex quinquefasciatus* mosquitoes and bird blood with variant-specific probes.

Additional file 2: Fig. S2. *Plasmodium relictum* SGS1 oocysts detected by chromogenic in situ hybridization applying an 18S variant-specific probe (SGS1-Pr1_ISH) to tissue sections of experimentally infected *Culex quinquefasciatus* (Cx4) (A–C). B, C Labeled oocysts (white arrows) were located in the midgut epithelium. Besides labeled oocysts, unstained, presumably degenerated oocysts (black arrows) were seen. Mg, midgut; ov, ovary; ep, epidermis; cu, cuticula. Scale bars: 500 µm (A) and 20 µm (B, C).

Additional file 3: Fig. S3. * Haemoproteus tartakovskyi* SISKIN1 oocysts detected by chromogenic in situ hybridization applying 18S variant-specific probes (SISKIN1-Pr3, A–C and SISKIN1-Pr2, D–G) to tissue sections of experimentally infected *Culicoides nubeculosus *(Cu11, 3 dpi). A–G Labeled oocysts (white arrows) were located within or in close association to the epidermis of the ventral abdomen of the vectors. Ov, ovary; mg, midgut; ep, epidermis; cu, cuticula. Scale bars: 100 µm (A, D) and 10 µm (B, C, E–G).

## Data Availability

All data generated or analysed during this study are included in this published article and its supplementary information files.

## Abbreviations

BCIP: 5-Bromo-4-chloro-3′-indolyphosphate *p*-toluidine salt
cDNA: Complementary DNA
CISH: Chromogenic in situ hybridization
dpi: Days post-infection
ES: Expansion segment(s)
NBT: Nitro-blue tetrazolium chloride
NRC: Nature Research Centre
qPCR: Quantitative PCR
