# Supplementary figures and images for: Haemoproteus tartakovskyi and Plasmodium relictum (Haemosporida, Apicomplexa) differentially express distinct 18S rRNA gene variants in bird hosts and dipteran vectors
Source: Parasit Vectors. 2025 Feb 20;18:63. doi: 10.1186/s13071-025-06696-0 (PMC11844136; doi:10.1186/s13071-025-06696-0)

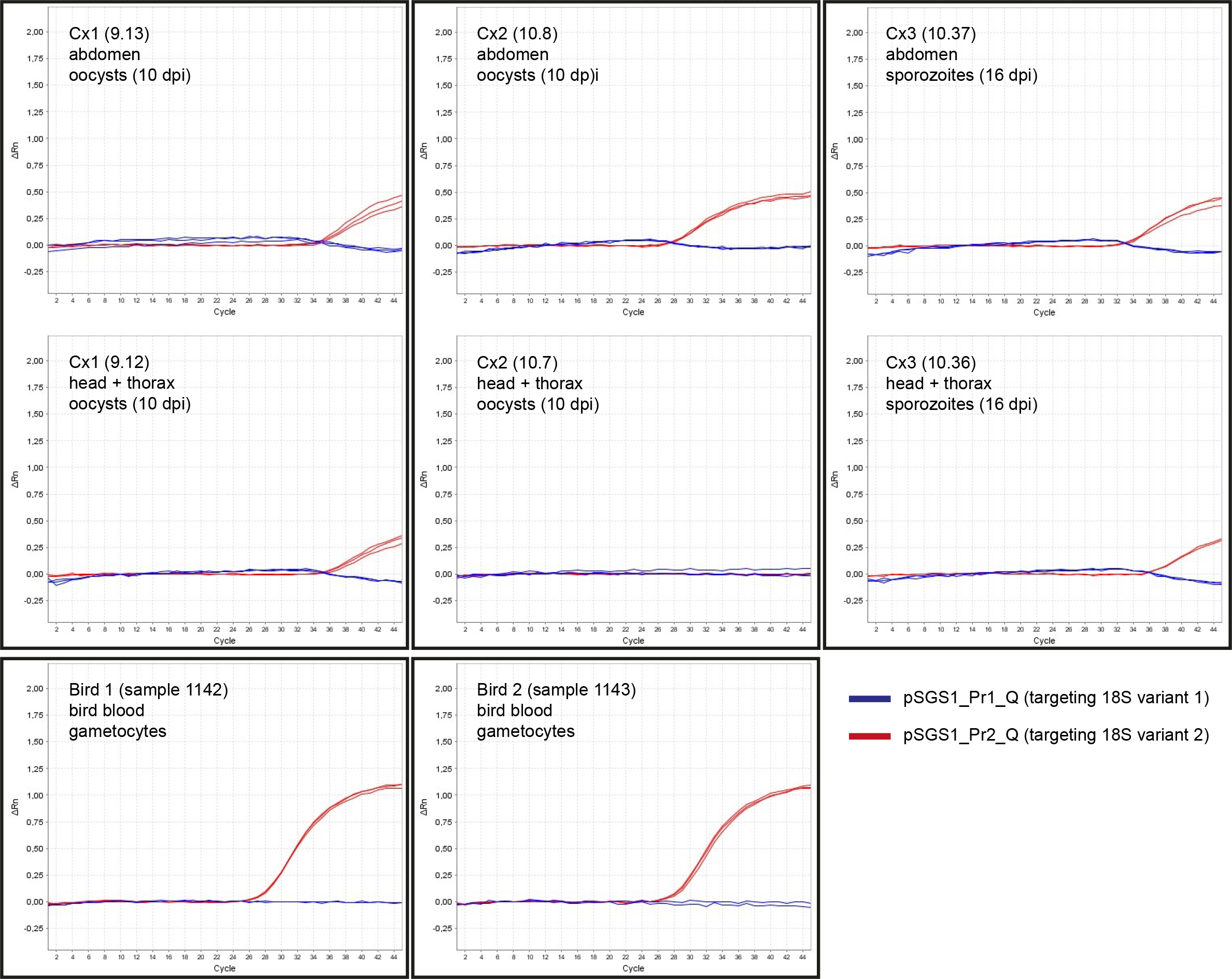

Supplement: Supplementary file 1 — Additional file 1: Fig. S1. Real-time quantitative PCRs (TaqMan qPCR assay) targeting the 18S rRNA of Plasmodium relictum SGS1 in Culex quinquefasciatus mosquitoes and bird blood with variant-specific probes. [file 13071_2025_6696_MOESM1_ESM.jpg]

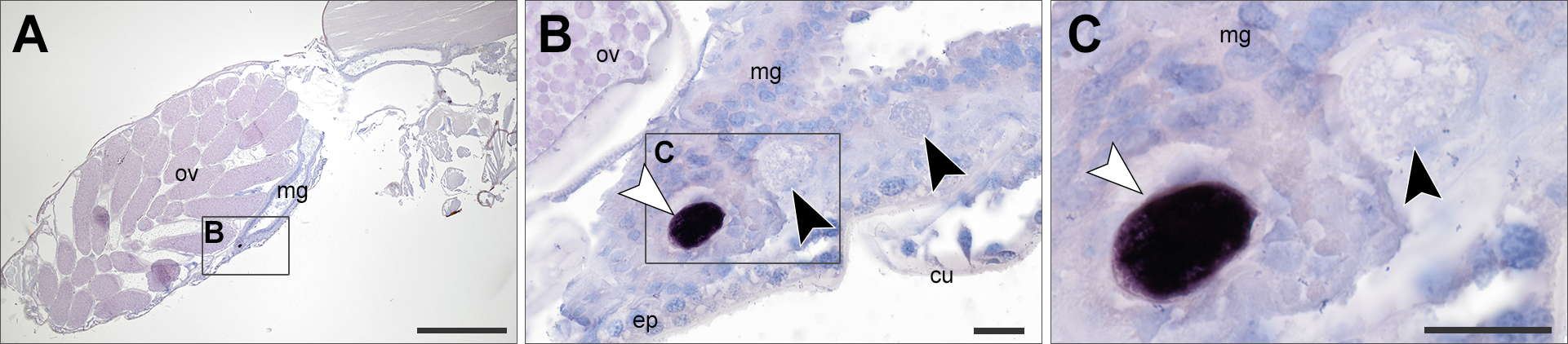

Supplement: Supplementary file 2 — Additional file 2: Fig. S2. Plasmodium relictum SGS1 oocysts detected by chromogenic in situ hybridization applying an 18S variant-specific probe (SGS1-Pr1_ISH) to tissue sections of experimentally infected Culex quinquefasciatus (Cx4) (A–C). B, C Labeled oocysts (white arrows) were located in the midgut epithelium. Besides labeled oocysts, unstained, presumably degenerated oocysts (black arrows) were seen. Mg, midgut; ov, ovary; ep, epidermis; cu, cuticula. Scale bars: 500 µm (A) and 20 µm (B, C). [file 13071_2025_6696_MOESM2_ESM.jpg]

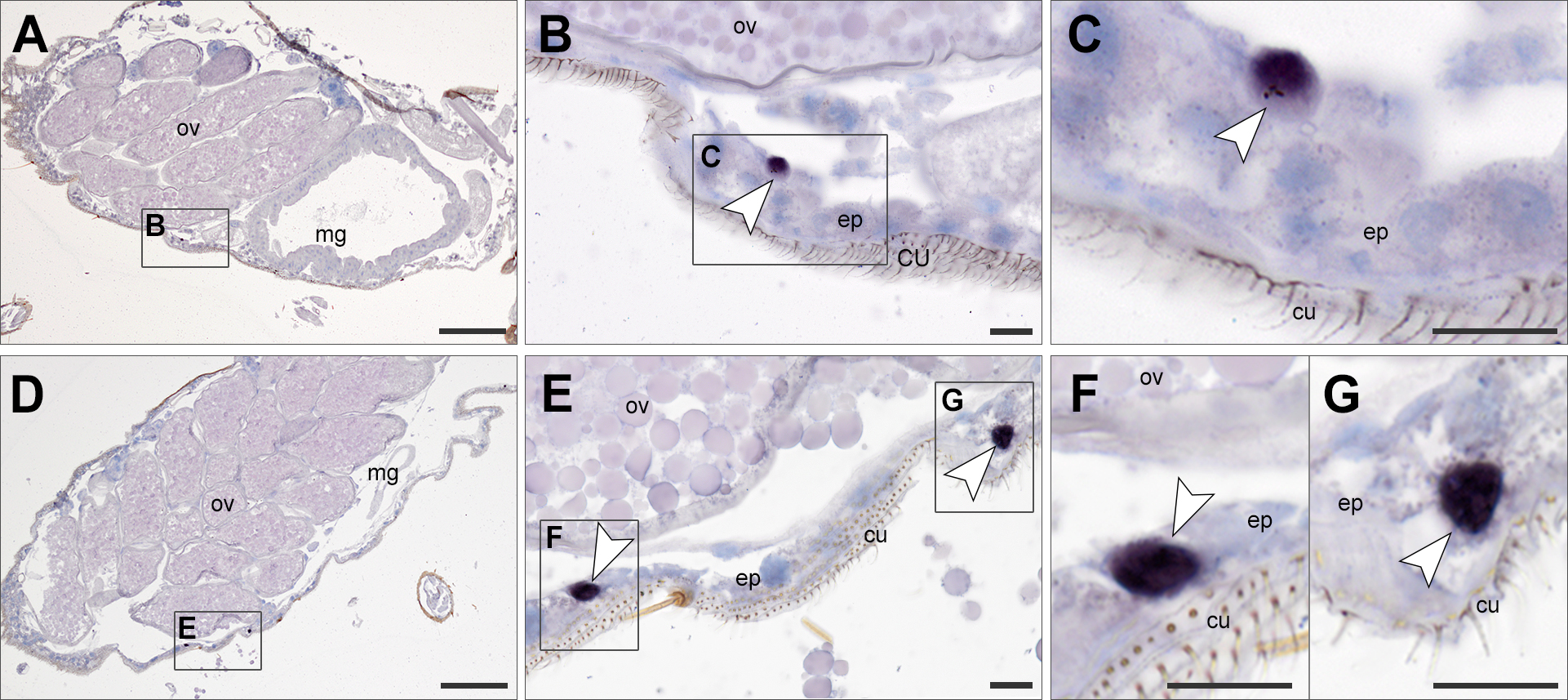

Supplement: Supplementary file 3 — Additional file 3: Fig. S3. Haemoproteus tartakovskyi SISKIN1 oocysts detected by chromogenic in situ hybridization applying 18S variant-specific probes (SISKIN1-Pr3, A–C and SISKIN1-Pr2, D–G) to tissue sections of experimentally infected Culicoides nubeculosus (Cu11, 3 dpi). A–G Labeled oocysts (white arrows) were located within or in close association to the epidermis of the ventral abdomen of the vectors. Ov, ovary; mg, midgut; ep, epidermis; cu, cuticula. Scale bars: 100 µm (A, D) and 10 µm (B, C, E–G). [file 13071_2025_6696_MOESM3_ESM.jpg]
